# Acute Postprandial Glycemic Responses to Breads Made from Five Commercial Wheat-Flour Formulations: A Small Exploratory Nonrandomized Within-Participant Crossover Study

**DOI:** 10.3390/nu18203396

**Published:** 2026-10-16

**Authors:** Selda Seçkiner, Aslıhan Atar-Uğurlu, Beyzanur Akgün, Elif Esin Çelik, Beyza Kaya, Zeynep Ayça Üneş, Su Özgür, Fatih Mehmet Velibeyoğlu, Murat Baş

**Affiliations:** 1 Department of Nutrition and Dietetics, Graduate School of Health Sciences, Acibadem Mehmet Ali Aydinlar University, Istanbul 34752, Türkiye; seldaseckiner@gmail.com; 2 Department of Nutrition and Dietetics, Faculty of Health Sciences, Istanbul Gelisim University, Istanbul 34310, Türkiye; aslihanatar@hotmail.com; 3 Independent Researcher, Istanbul 34000, Türkiye; dyt.beyzanurakgun@gmail.com (B.A.); elifesincelik@gmail.com (E.E.Ç.); beyzakaya60@icloud.com (B.K.); uneszeynep70@gmail.com (Z.A.Ü.); 4 Translational Pulmonary Research Center (EgeSAM), Ege University, Izmir 35100, Türkiye; suozgur35@gmail.com; 5 Council of Forensic Medicine, Ministry of Justice, Istanbul 34196, Türkiye; ctffatih@yahoo.com; 6 Department of Nutrition and Dietetics, Faculty of Health Sciences, Acibadem Mehmet Ali Aydinlar University, Istanbul 34752, Türkiye

**Keywords:** postprandial glycemia, bread, purple flour, einkorn, Karakılçık, durum wheat

## Abstract

**Background/Objectives**: Glycemic responses to bread vary with flour composition and processing; this study compared white bread with four specialty wheat-flour breads consumed in Türkiye. **Methods**: This nonrandomized five-condition within-participant crossover study included 12 healthy adults. Test flours were a purple-labeled refined flour formulated with fruit and vegetable extracts and ascorbic acid (not purple wheat), wholemeal einkorn, refined yellow durum, and wholemeal Karakılçık landrace. White bread was consumed first, then the test breads in two fixed, partially balanced sequences. Portions provided 50 g available carbohydrate from manufacturer-declared composition. Capillary glucose was measured at 0, 30, 60, 90, and 120 min; the primary outcome was total incremental area under the curve (iAUC). **Results**: Purple-flour bread had 32% lower total iAUC (Holm-adjusted *p* = 0.035) and early iAUC (0–60 min; *p* = 0.041) than white bread. The early contrast held in the Wilcoxon test (*p* = 0.049), whereas interpretation of the total iAUC contrast depended on the model: it reached significance in neither rank-based Wilcoxon (*p* = 0.064) nor log-transformed (*p* = 0.058) analyses. In exploratory comparisons, purple-flour bread had lower early iAUC than the other test breads and lower total iAUC than yellow durum (all Holm-adjusted *p* ≤ 0.016). **Conclusions**: The purple formulation was associated with lower early glycemic exposure, einkorn with intermediate and yellow durum with the highest responses; Karakılçık did not differ significantly from white bread. Higher declared fiber content was not consistently associated with lower acute glycemic responses in the products tested. Findings apply only to the formulations tested and require confirmation in randomized, counterbalanced studies with verified composition.

## 1. Introduction

Foods containing similar amounts of carbohydrates can produce markedly different postprandial blood glucose responses. The glycemic index (GI), developed by Jenkins et al., quantifies this difference and reflects not only the total carbohydrate content of a food but also the rate at which the carbohydrate is digested and absorbed [1,2]. Although the long-term health effects of high-GI diets cannot be inferred from a single acute glycemic response, a large prospective study involving 137,851 participants from 20 countries reported that a higher dietary GI was associated with increased risks of major cardiovascular events or death in individuals with and without preexisting cardiovascular disease [3]. Updated meta-analyses of prospective cohorts revealed a dose–response relationship between dietary GI or GL and the risk of type 2 diabetes mellitus [4]. Therefore, frequently consumed carbohydrate sources warrant evaluation in terms of not only nutrient composition but also the acute glycemic response they elicit.

Bread is a staple carbohydrate source in many dietary patterns; however, it cannot be assumed that all breads share similar glycemic properties. In a survey involving 1766 adults in Türkiye, white bread was the most frequently preferred type, and bread supplied approximately one-quarter of daily energy intake, highlighting the local relevance of comparing commonly used and traditional flours [5]. The postprandial glycemic impact of bread is determined by interrelated factors, including wheat cultivar, refinement degree, particle size, amylose-to-amylopectin ratio, starch gelatinization, fiber and protein contents, fermentation, baking conditions, and final crumb structure. Breads of similar nutrient composition but more compact structure can lower the initial rate of glucose appearance and peak response compared with more porous breads [6]. Preservation of coarse particle structure can also lower postprandial glycemia by restricting enzymatic access to starch [7]; however, this advantage may diminish during normal mastication [8]. Furthermore, glycemic responses to bread show a substantial interindividual component [9,10]. Therefore, general descriptors such as “wholegrain” or “traditional” are insufficient on their own to predict glycemic impact.

The genetic characteristics of wheat and the resulting starch structure may also influence glycemic responses. In healthy adults, bread made from high-amylose wheat lowered postprandial glucose iAUC by approximately 39% compared with conventional low-amylose wheat bread [11]. Similarly, high-amylose barley bread reduced the 4 h glucose iAUC by 28% [12]. A more recent crossover trial remotely conducted with continuous glucose monitoring indicated that this effect depends on bread formulation and individual response characteristics [13]. Pigmented cereals have attracted similar interest as their anthocyanins may act on carbohydrate-digesting enzymes and starch digestibility [14,15]. However, these effects seem to depend on the pigment source, food matrix, and dose consumed. Wheat bread fortified with an anthocyanin-rich extract exhibited a reduced GI, whereas the same extract consumed within a mixed meal produced no comparable improvement in the postprandial glucose response [16,17]. An 8-week human study comparing whole-purple-wheat products with regular whole-wheat products also reported limited overall metabolic differences between the two wheats [18].

Ancient and landrace wheats form another part of this discussion. Einkorn (*Triticum monococcum* L.), a diploid ancient species, differs from modern bread wheats in terms of grain, protein, and carotenoid characteristics [19]. A randomized crossover trial in humans associated einkorn bread with more favorable postprandial glucose, insulin, and satiety responses than refined modern-wheat breads [20]. Contrarily, a study comparing breads formulated from different wheat evolutionary populations showed no significant differences in glucose iAUC or peak glucose response [21]. Karakılçık (*Triticum turgidum* L. subsp. *durum*), a durum wheat landrace native to Türkiye, has been characterized in terms of its physical, chemical, and technological properties [22]. However, to our knowledge, its postprandial glycemic response has not yet been evaluated in humans. Furthermore, breads made from purple flour, einkorn, yellow durum, and Karakılçık have not been directly compared in the same participants, and early-phase glycemic responses have rarely been evaluated separately.

Two further points matter for the interpretation of comparisons between commercially available flours. First, retail flours are products rather than genotypes: they may differ simultaneously in wheat species, milling degree, particle size, fiber and protein content, and permitted additives, and some are formulated with ingredients that are not derived from wheat at all. The present study therefore evaluates commercial wheat-flour formulations as they reach the consumer rather than isolated wheat-variety effects. Second, the directional hypotheses rested on distinct and partly independent mechanisms. Lower glycemic exposure was expected for the purple-labeled formulation because the added anthocyanin-rich fruit and vegetable extracts may inhibit α-amylase and α-glucosidase and restrict starch accessibility within the bread matrix [14,15,17]; for einkorn because its distinctive protein and starch characteristics and denser crumb may slow starch digestion [6,19,20]; and for Karakılçık because it is a wholemeal landrace flour with a high declared fiber content and a coarser particle structure, both of which can restrict enzymatic access to starch [7,22]. Yellow durum flour, in contrast, was refined and low in declared fiber and was therefore not expected to lower glycemic exposure relative to the refined white reference.

This study aimed to compare acute postprandial glycemic responses to white, purple-flour, einkorn, yellow durum, and Karakılçık breads in healthy adults using a small, exploratory, nonrandomized within-participant crossover design. Because the tested products were finished commercial formulations rather than isolated wheat genotypes, the comparison was framed at the level of the formulation as sold. It was hypothesized that bread type would influence total and early glycemic responses and that purple flour, einkorn, and Karakılçık breads would produce lower glycemic exposure than the white-bread reference, for the mechanistic reasons outlined above, whereas the refined, low-fiber yellow durum bread was not expected to do so.

## 2. Materials and Methods

### 2.1. Study Design

This was a single-group, five-condition, nonrandomized, within-participant crossover study. Each participant consumed the white-bread reference and four test breads (purple flour, einkorn, yellow durum and Karakılçık) in separate sessions, with an interval of at least 1 week. The primary outcome was the total incremental area under the curve (iAUC) for capillary glucose over 0–120 min, geometrically calculated. The secondary outcomes included early iAUC (0–60 min), *C*_max_, and *T*_max_.

The protocol was developed with reference to international methods for assessing postprandial glycemic responses [2,23]. It deviated from standard GI methodology in that (i) samples were obtained at 0, 30, 60, 90, and 120 min, (ii) one fasting reading was obtained per session, (iii) each postprandial time point was represented by one reading, and (iv) the white-bread reference was administered only once per participant. Therefore, the study evaluated comparative acute glycemic responses rather than standard GI values.

The protocol was approved by the Non-Interventional Clinical Research Ethics Committee of Istanbul Medipol University on 14 November 2024 (approval no. 10840098-202.3.02-6985). Written informed consent was obtained before any study-specific procedure, and the study was conducted in accordance with the principles of the Declaration of Helsinki. The study was not registered in a public clinical trial registry. The outcome hierarchy, the geometric iAUC calculation, and the Holm-adjusted contrasts against white bread were defined before the data were analyzed but were not publicly preregistered. Consequently, the distinction between prespecified and post hoc elements cannot be independently verified by readers, and selective reporting cannot be formally excluded. To mitigate this, every analysis that was performed is reported in Section 3, including contrasts that did not reach statistical significance and sensitivity analyses that did not support the primary result; no outcome or comparison was omitted.

### 2.2. Participants

Participants were healthy adults aged 20–40 years who were recruited via snowball sampling. Eligibility required a body mass index (BMI) of 18.5–29.9 kg/m^2^, no self-reported metabolic or other chronic diseases, no regular medication use, and provision of written informed consent. Pregnant or lactating individuals were not enrolled. None of the participants reported engagement in regular physical activity. Smoking status and habitual alcohol consumption were not recorded. Of the 13 eligible participants enrolled, 1 withdrew for personal reasons, whereas 12 completed all five conditions and were included in the full analysis set. Participant-level pairing was preserved in all calculations and hypothesis tests.

No prospective sample-size calculation based on an expected between-bread difference was performed. The sample size was based on the international standard, which specifies at least 10 participants for GI determination [2], and interlaboratory comparison studies [24]; this standard was used as a practical minimum even though the present study did not determine GI values, and it is acknowledged that a standard developed for GI testing is not a substitute for a power calculation targeted at the comparisons actually made here. With 12 participants, the minimum detectable effect size for a two-sided paired-samples *t* test at α = 0.05 and 80% power was *d*_z_ ≈ 0.89; this value represents the smallest paired effect that the available sample could detect rather than an effect size specified in advance. This value was calculated for a single, unadjusted two-sided comparison and does not take account of the Holm adjustment applied within the families of four or six contrasts; because multiplicity adjustment lowers the effective significance threshold for each individual contrast, the minimum detectable effect in the analyses actually reported here is larger than *d*_z_ ≈ 0.89. This figure should therefore be read only as an approximate indication of the limited statistical power of the available sample and not as a precise power estimate for any individual contrast. Therefore, the study was powered to detect only large paired effects, and all inferential results should be read in that light.

### 2.3. Data Collection

Data were collected between July and October 2025. Sessions were conducted in the participants’ homes to avoid travel-related exertion and acute stress. Research team members weighed and served the portions, monitored the consumption, and performed every capillary glucose measurement using the same protocol; the participants did not measure their own glucose. Before each baseline measurement, the participants rested for 15 min while seated and remained seated during follow-up. The same pre-session fasting and activity restrictions were applied in all conditions.

### 2.4. Flours and Bread Production

White bread prepared from refined wheat flour served as the reference. All five flours were commercially available products of a single manufacturer (Nora Un, Ankara, Türkiye) and commercially purchased by the research team. According to the manufacturer labels, einkorn and Karakılçık flours were wholemeal, whereas the white reference, purple, and yellow durum flours were refined “special purpose wheat flours.” The purple product was wheat flour formulated using ascorbic acid (E-300) and extracts of blackberry, sour purple mulberry, blueberry, pomegranate, sour cherry, eggplant, and purple onion skin. Yellow flour was produced from durum wheat (*Triticum turgidum* subsp. *durum*) and Karakılçık flour from a Turkish landrace of the same species. All four test flours, but not the white reference flour, contained E-300. The label did not report the amount of E-300 or of the plant extracts; thus, their contributions could not be separated from other formulation differences. The product identity, label designation, declared ingredient list, milling status, and additive information available for each of the five flours are summarized in Table 1. The quantitative amounts of E-300 and of the individual plant extracts, the wheat cultivar, and the extraction (ash) rate of the refined flours were not disclosed by the manufacturer and could not be obtained on request.

All five breads were produced under a single common protocol so that the formulations differed only in the flour used: the water-to-flour ratio, the yeast-to-flour ratio, the salt amount, the mixing endpoint, the resting and final proofing times, and the oven temperature and baking time were identical across conditions, and every preparation was carried out by the same research team members in the same kitchen with the same equipment. Each recipe used the relevant flour, water, fresh yeast, and 6 g salt. Fresh yeast was added at 4.2% of the flour weight in all formulations. Water was set at 58.3% of the flour weight, excluding water intrinsic to fresh yeast. As flour quantities differed among recipes, fresh yeast amounts ranged from 21.0 to 25.2 g, whereas salt ranged from 1.0% to 1.2% of flour weight (baker’s percentages; Table 1). The ingredients were mixed until a smooth consistency was achieved and then allowed to stand for 15–20 min. Subsequently, the dough was divided into the pre-baking portions listed in Table 2 and shaped individually. Then, the portions underwent final proofing for 30 min at room temperature before being baked individually in an oven at 180 °C for 45 min. The internal bread temperature was not measured. The portions were baked on the morning of each test session, allowed to cool completely, and then served. The same type of fresh yeast was used in all recipes; no sourdough was added.

Each bread type was prepared in four separate batches. Table 2 presents the full recipe composition. Three individually weighed study portions were taken from each batch, and the remaining dough was not used in the study. Preparations were performed by the research team members using the same stated recipe, temperature, and time.

### 2.5. Portioning and Available Carbohydrates

Energy and macronutrient calculations were based on manufacturer label declarations and the flour quantity in each recipe (Table 3). The declared carbohydrate values excluded dietary fiber and were directly used as available carbohydrate. No further fiber subtraction was applied:

*Available carbohydrate (g/100 g flour) = manufacturer-declared carbohydrate (g/100 g flour)*

The pre-baking dough-portion weight was calculated to allocate flour providing 50.0 g of label-derived available carbohydrate:

*Dough portion (g) = [50/available carbohydrate from flour in the recipe (g)] × total pre-baking recipe mass (g)*

To calculate the total pre-baking recipe mass, water volume was converted to mass assuming a 1 g/mL density. Individual dough portions were weighed to the calculated targets in Table 2. Each portion was separately baked and served in its entirety after cooling; thus, moisture loss did not change the amount of flour allocated to the portion. Dietary fiber was not declared for the white reference flour and was therefore recorded as not reported rather than assumed to be zero. The manufacturer-declared carbohydrate values excluded dietary fiber and were directly used to calculate portions providing an estimated 50 g of available carbohydrate. The carbohydrate dose and other nutrient amounts were label-based estimates; neither flour nor baked-bread composition was verified by laboratory analysis.

### 2.6. Experimental Protocol

The participants attended each test session after a 12 h overnight fast. On the preceding evening, they were asked to consume their habitual evening meal; avoid alcohol and caffeine, unaccustomed physical activity, and nighttime snacking; and obtain sufficient sleep. The evening meal and compliance variables were not recorded. After 15 min of seated rest and baseline glucose measurement, the relevant portion was consumed within 10–12 min with 250 mL water. The participants remained seated for 120 min, consumed no other food or energy-containing beverage, and refrained from physical activity. Each condition was administered once.

White bread was administered in period 1 to every participant. The four test breads followed one of two fixed sequences without formal randomization: purple flour → einkorn → yellow durum → Karakılçık in six participants and yellow durum → Karakılçık → einkorn → purple flour in the remaining six. Research team members assigned participants nonrandomly to a sequence according to scheduling feasibility. Consecutive sessions were separated by at least 1 week. As bread appearance and texture differed, participant blinding was not feasible. The fixed white-first design prevents the separation of a white-bread effect from a period-1 effect.

### 2.7. Capillary Blood Glucose Measurement

Capillary blood glucose was measured via fingertip sampling immediately before consumption (0 min) and at 30, 60, 90, and 120 min. The same CONTOUR^®^ PLUS glucometer and compatible CONTOUR^®^ PLUS strips from one lot (Ascensia Diabetes Care, Basel, Switzerland) were used in all sessions. Blood glucose monitoring systems of this platform have been reported to meet the ISO 15197:2013 accuracy criteria [25]. Researchers performed one measurement at each time point; duplicate readings were not obtained. The first blood drop was discarded, and the second was applied to the strip. Measurements were performed with the participants seated after the 15 min rest period. The results were expressed in mg/dL.

### 2.8. Outcome Calculation

For each participant and bread condition, the time-specific incremental glucose value was calculated by subtracting the fasting value of that session from the measured glucose value:

ΔG_t_ = G_t_ − G_0_

The primary outcome was the geometric iAUC between 0 and 120 min, whereas the secondary outcome was the early-phase iAUC (0–60 min). iAUC values were obtained using the linear trapezoidal method, including only the area above the fasting level. Where the curve crossed the fasting line between two consecutive time points, the crossing time was determined by linear interpolation, and only the geometric segment above the line was included in the integral; areas below the fasting level were neither subtracted nor offset against the positive area. This approach is consistent with the international standard [2].

*C*_max_ denotes the highest incremental glucose value observed at the scheduled measurement points over 0–120 min, and *T*_max_ denotes the scheduled time point at which this value was observed. Where equal maxima occurred, the earlier time point was used.

### 2.9. Statistical Analysis

Continuous variables were expressed as mean ± standard deviation or median [interquartile range, IQR]. All analyses preserved participant-level pairing, and the normality of paired differences was evaluated using the Shapiro–Wilk test. Differences in total iAUC, early iAUC, *C*_max_, and fasting glucose across the five bread conditions were also evaluated via one-way repeated-measures analysis of variance (ANOVA). Greenhouse–Geisser-corrected results and partial eta squared (η_p_^2^) were reported. Friedman tests were employed as nonparametric sensitivity analyses. In the primary comparisons, each test bread was evaluated against white bread using paired-samples *t*-tests with Holm adjustment within each outcome. The results are expressed as mean paired differences with nominal 95% confidence intervals (CIs) and Cohen’s *d*_z_. Holm-adjusted Wilcoxon signed-rank tests and log-transformed total iAUC analyses were conducted as sensitivity analyses. All total iAUC values were positive; therefore, natural logarithmic transformation was applied without addition of a constant. Glucose measurements were complete at all five time points under all five conditions for the 12 participants who completed the study, and imputation was not performed. In the exploratory analyses, the four test breads were compared after exclusion of the white-bread condition. Overall and pairwise differences were evaluated via repeated-measures ANOVA and Holm-adjusted paired *t*-tests, respectively, with Wilcoxon and log-transformed total iAUC analyses used to assess sensitivity. Holm adjustment covered four white-reference or six test-bread contrasts within each outcome and analytical approach. Purple-minus-yellow-durum total iAUC differences were also descriptively summarized by sequence. *T*_max_ was analyzed using the Friedman test. Figures display means with within-participant 95% CIs calculated using Cousineau normalization and the Morey correction. All tests were two-sided with α = 0.05. Analyses were conducted using IBM SPSS Statistics for macOS, version 30 (IBM Corp., Armonk, NY, USA). The analyses were organized in a fixed hierarchy. The comparison of each test bread with white bread for total iAUC (0–120 min) was the primary analysis; the corresponding contrasts for early iAUC and *C*_max_ were secondary; and all comparisons among the four test breads were exploratory and hypothesis-generating. Holm adjustment was applied within, but not across, these families, so the exploratory results do not carry the same evidentiary weight as the primary contrast and are reported for completeness rather than as confirmatory findings. Confidence intervals are nominal, meaning that they were not widened to account for multiplicity and therefore do not correspond exactly to the Holm-adjusted *p*-values: a nominal interval excluding zero should not be read as statistical significance after adjustment. The Holm-adjusted *p*-value takes precedence for inference, whereas the interval conveys the magnitude and precision of the estimated difference.

## 3. Results

### 3.1. Participant Characteristics and Baseline Comparability

The 12 individuals (8 women and 4 men) who completed the study were aged 21–25 years. Height ranged from 165 to 186 cm and BMI from 19.7 to 29.5 kg/m^2^ (Table 4). The mean fasting capillary glucose across sessions was 95.1 ± 5.6 mg/dL and did not differ across conditions (*F* (2.09, 23.01) = 0.83; *p* = 0.454; η_p_^2^ = 0.070). Session-specific means ranged from 93.5 mg/dL for white bread to 96.2 mg/dL for Karakılçık (Table 5). No adverse events or undesirable effects were observed or reported during any test session.

### 3.2. Postprandial Glucose Curves

**Figure 1:**
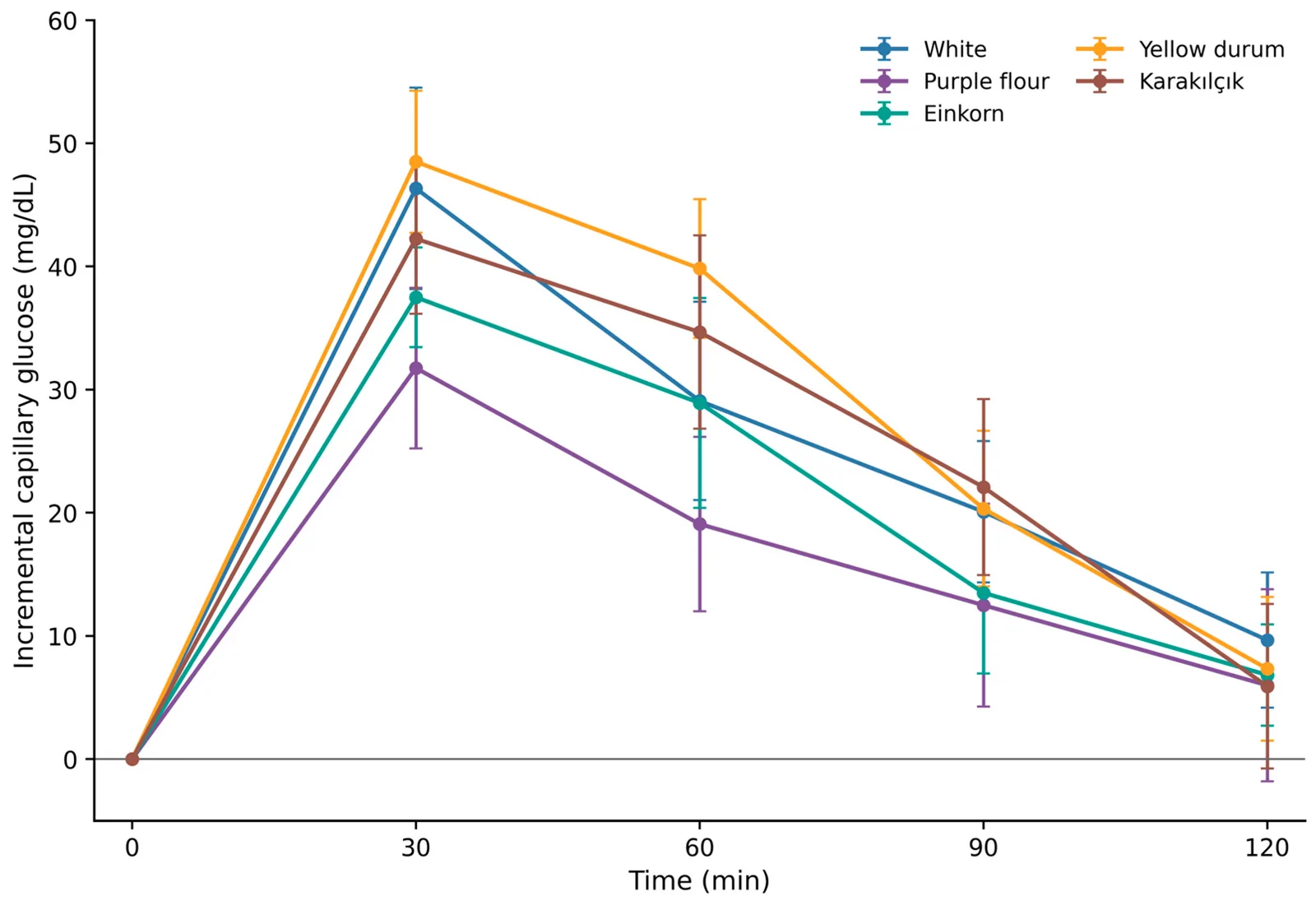
Postprandial incremental capillary blood glucose curves by bread type (*n* = 12). Symbols show means; error bars denote within-participant 95% confidence intervals calculated using Cousineau normalization and the Morey correction across the five bread conditions separately at each time point. Incremental glucose was calculated compared with the fasting value in the same session.

The mean incremental glucose responses peaked at 30 min under all bread conditions and progressively declined toward 120 min (Figure 1). The highest mean increments at 30 min were observed for yellow durum (48.5 mg/dL) and white bread (46.3 mg/dL), whereas the lowest was observed for purple flour bread (31.8 mg/dL). The purple flour curve remained numerically lowest at 30, 60, and 90 min, whereas separation between conditions was markedly attenuated by 120 min. These time-specific means differ from *C*_max_, which summarizes individual observed peaks.

### 3.3. Primary and Secondary Outcomes

Bread type had a significant main effect on total iAUC (*F* (2.93, 32.27) = 5.09; *p* = 0.006; η_p_^2^ = 0.316), early iAUC (*F* (2.18, 23.94) = 7.50; *p* = 0.002; η_p_^2^ = 0.405), and *C*_max_ (*F* (2.72, 29.88) = 4.51; *p* = 0.012; η_p_^2^ = 0.291) (Table 5). Purple-flour bread produced the lowest mean total iAUC, early iAUC, and *C*_max_ and yellow durum bread the highest. *T*_max_ clustered at a median of 30 min under all conditions and did not differ between breads (Friedman χ^2^ = 5.00; df = 4; *p* = 0.287); all 12 participants peaked at 30 min with white bread, and 9 to 10 participants did so under the other conditions.

**Figure 2:**
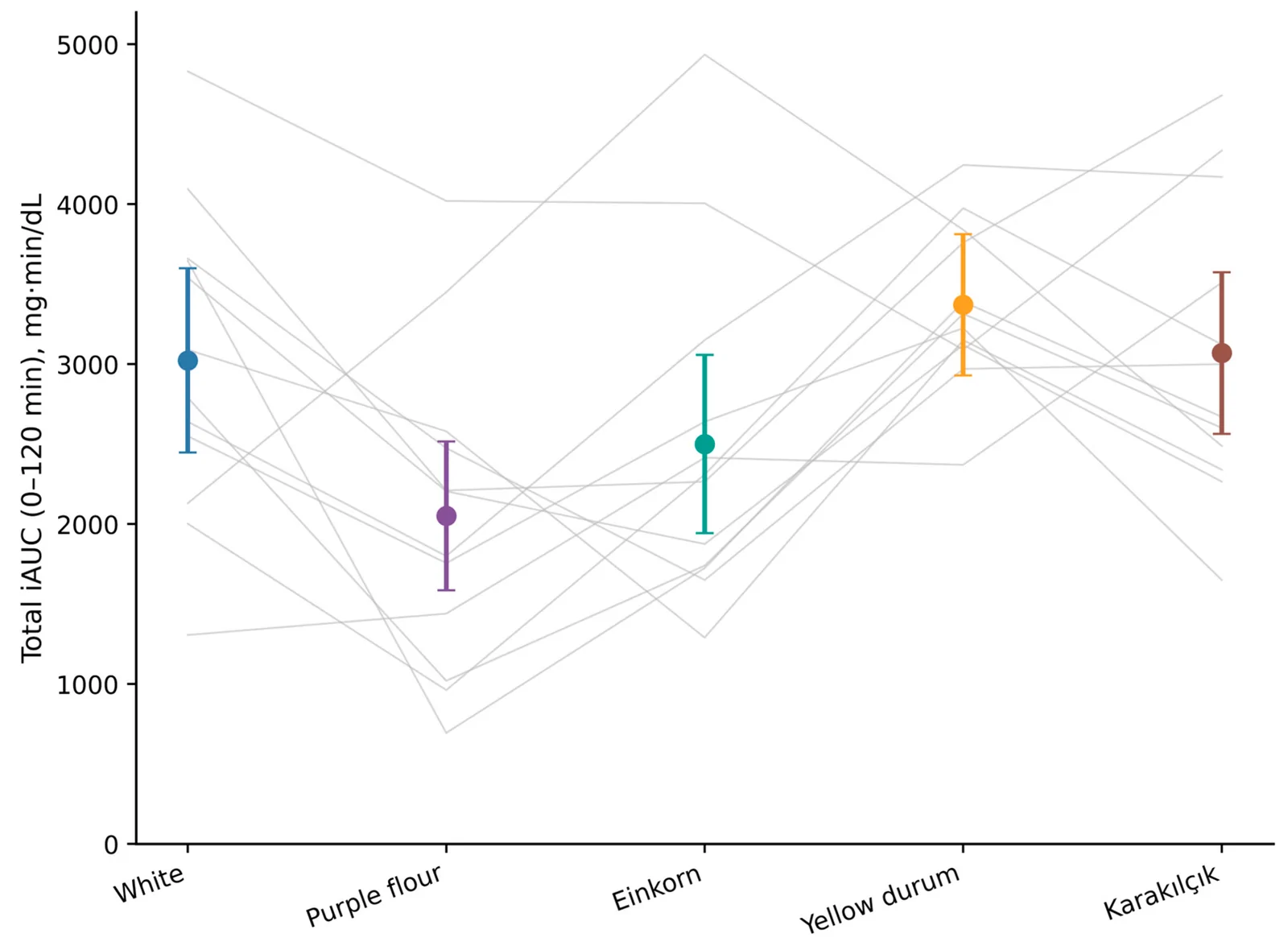
Total geometric iAUC by bread type with participant-level pairing (*n* = 12). Gray lines connect the five values of each participant; symbols show means with within-participant 95% confidence intervals calculated via Cousineau normalization and the Morey correction.

Participant-level trajectories illustrate substantial interindividual variability in total iAUC and the lower group mean for purple-flour bread (Figure 2).

### 3.4. White-Bread Reference Comparisons and Sensitivity Analyses

In the primary analysis of total iAUC and the secondary analyses of early iAUC and *C*_max_, after Holm adjustment within each family of four contrasts, purple-flour bread had lower total iAUC (mean difference −972.2 mg·min/dL; nominal 95% CI −1645.0 to −299.4; Holm *p* = 0.035; Cohen’s *d*_z_ = −0.92) and early iAUC (−587.5 mg·min/dL; nominal 95% CI −1005.4 to −169.6; Holm *p* = 0.041; *d*_z_ = −0.89) than white bread (Table 6). Its *C*_max_ was also lower, but not after Holm adjustment (*p* = 0.082). No other test bread significantly differed from white bread in these reference contrasts.

The secondary early-iAUC contrast for purple flour remained significant in the Wilcoxon sensitivity analysis (Holm *p* = 0.049). The primary total iAUC contrast remained directionally consistent but did not reach statistical significance in either the Wilcoxon (Holm *p* = 0.064) or log-scale (Holm *p* = 0.058) analyses, indicating sensitivity to analytical assumptions. In the descriptive by-sequence summary, the purple-minus-yellow-durum total iAUC difference was negative in both fixed test-bread sequences, indicating that the direction of this contrast did not depend on the order in which the two breads were consumed. Because white bread was always consumed in period 1, no corresponding by-sequence comparison is possible for the white-bread contrasts, and these therefore remain confounded with period.

### 3.5. Exploratory Comparisons Among the Four Test Breads

In the exploratory, hypothesis-generating analysis restricted to the four test breads, bread type affected the total iAUC (Greenhouse–Geisser *F* (2.56, 28.15) = 7.04; *p* = 0.002; η_p_^2^ = 0.390), early iAUC (*F* (2.21, 24.35) = 14.09; *p* < 0.001; η_p_^2^ = 0.562), and *C*_max_ (*F* (2.22, 24.45) = 6.66; *p* = 0.004; η_p_^2^ = 0.377). Following Holm adjustment across six contrasts, the purple-flour bread had lower total iAUC than the yellow durum bread (mean difference −1319.8 mg·min/dL; nominal 95% CI −2050.3 to −589.4; *t*-test *p* = 0.013; Wilcoxon *p* = 0.032; *d*_z_ = −1.15). The same contrast remained significant on the log scale (Holm *p* = 0.020). Although the difference in total iAUC between purple-flour and Karakılçık breads was large (−1018.3 mg·min/dL; *d*_z_ = −0.84), it did not remain statistically significant following multiplicity adjustment (*t*-test *p* = 0.066; Wilcoxon *p* = 0.107; log-scale *p* = 0.055). Notably, the purple-flour bread had lower early iAUC than einkorn (Holm *p* = 0.016), yellow durum (*p* < 0.001), and Karakılçık (*p* = 0.016), with the differences supported by Holm-adjusted Wilcoxon tests (*p* ≤ 0.027). Einkorn bread produced a lower early iAUC (0–60 min) than yellow durum bread (Holm-adjusted *p* = 0.001 for the paired *t*-test and *p* = 0.010 for the Wilcoxon signed-rank test). The difference in total iAUC between the einkorn and yellow durum breads was not statistically significant following Holm adjustment in the paired *t*-test or Wilcoxon analyses (*p* = 0.066 and *p* = 0.063, respectively) but was significant in the log-transformed paired *t*-test sensitivity analysis (Holm-adjusted *p* = 0.032). For *C*_max_, purple flour and einkorn had lower values than yellow durum (Holm *t*-test *p* = 0.008 and *p* = 0.017; Wilcoxon *p* = 0.023 and *p* = 0.034, respectively). These comparisons were not prespecified as confirmatory analyses and are reported in Table 7.

## 4. Discussion

This nonrandomized within-participant crossover study compared acute glycemic responses to five breads in 12 healthy adults and, to our knowledge, is the first to conduct human postprandial glycemic evaluation of Karakılçık. Purple-flour bread was associated with approximately 32% lower total and early iAUC than white bread in the main analysis. The early response was supported by the Wilcoxon analysis, whereas the total iAUC contrast was sensitive to analytical assumptions. In exploratory comparisons among the four test breads, purple flour was associated with lower early iAUC than each of the other three breads and lower total iAUC than yellow durum. These findings identify a formulation-specific difference in the acute glycemic response, with the clearest evidence concerning early glycemic exposure. Because the design was nonrandomized and the reference bread was always consumed first, these contrasts describe associations observed under the tested conditions rather than established causal effects of the flours.

The interpretation depends on the tested product. Purple flour is a refined special-purpose wheat flour containing added fruit and vegetable extracts, rather than flour from a purple-wheat genotype. All test flours contained ascorbic acid (E-300), whereas the white reference did not; the amounts of E-300 and plant extracts were undeclared. Therefore, the observed difference could not be attributed to the added anthocyanin-rich extracts, ascorbic acid, or the flour itself as these factors were confounded within a single commercial product. The findings are directionally consistent with the lower GI reported for bread fortified with anthocyanin-rich black rice extract [16], whereas longer-term comparisons of whole purple and regular wheat have demonstrated limited metabolic differences [18]. Polyphenols may affect carbohydrate-digesting enzymes and starch accessibility in matrix-dependent ways [14,15,17]. However, anthocyanin content, total phenolics, starch composition, in vitro digestibility, and other mechanistic markers were not measured in the present study. These mechanisms are therefore cited only as context from the existing literature and are not advanced as explanations of the present findings. Moreover, the five flours differed simultaneously in milling degree, declared fiber and protein content, and additive composition, and the resulting breads may have differed in crumb structure; the observed response therefore cannot be attributed to any single component and should be read as a property of the finished commercial formulation as a whole.

The total and early iAUC were approximately 17% and 15% lower for einkorn than for white bread, respectively; however, the white-reference comparisons were not significant following Holm adjustment. Einkorn produced lower early iAUC and *C*_max_ than yellow durum; the difference in total iAUC between them was significant only in the log-scale sensitivity analysis. This pattern is partly consistent with a trial reporting more favorable postprandial responses to einkorn bread [20], although another trial comparing breads made from different wheat evolutionary populations found no significant differences in glucose iAUC or peak glucose levels [21]. The wide confidence intervals in the present study do not support an equivalence conclusion.

Yellow durum and Karakılçık belong to the same wheat species but differ in genotype and declared milling degree. Yellow durum produced the highest mean total and early glycemic responses, whereas Karakılçık showed responses comparable to those of white bread; yellow durum and Karakılçık did not significantly differ in the pairwise analyses. Purple flour produced lower early iAUC than Karakılçık; however, their total iAUC difference did not survive Holm adjustment. Therefore, this first human evaluation did not detect a statistically significant lower acute response for Karakılçık under the tested conditions. This absence of a significant difference must not be read as evidence of equivalence: with 12 participants, the nominal 95% confidence interval for the Karakılçık-minus-white total iAUC contrast extended from −659.4 to 751.6 mg·min/dL, so differences in a magnitude that could still be nutritionally relevant cannot be excluded. An ancient or traditional grain name alone does not establish a glycemic advantage: milling, particle size, fermentation, and bread structure can influence the response [6,7,8,19]. These factors may help explain why the relatively high declared fiber content of Karakılçık did not translate into a clearly lower response, although their individual contributions were not evaluated. Among the four test flours, for which fiber content was declared, higher declared fiber content was not consistently associated with lower acute glycemic responses: the two wholemeal flours with the highest declared fiber content (einkorn and Karakılçık, 13.0 g/100 g) did not produce the lowest responses, whereas the refined purple formulation (2.03 g/100 g) did. No formal analysis of declared fiber content as a predictor of the glycemic response was performed, and with only five products this observation is descriptive rather than inferential.

The flour in each portion was calculated to provide 50 g of available carbohydrate, whereas other nutrient amounts differed. The estimated flour-derived protein ranged from 7.1 g in white bread to 9.3 g in yellow durum; einkorn and Karakılçık each contributed approximately 8.6 g of protein and 9.3 g of fiber. These differences are part of the tested formulations and may influence postprandial responses. A one-meal glycemic difference does not establish long-term clinical benefit, and broader evidence on carbohydrate quality remains relevant when assessing the nutritional value of breads [26].

The strengths of this study include completion of all five conditions by every participant, preservation of participant-level pairing, partial balancing of the test-bread sequences, and researcher-performed measurements using a single meter and strip lot. Multiplicity adjustment and sensitivity analyses helped assess the uncertainty of the observed differences.

The most important limitation concerns the primary comparison itself. White bread was administered in period 1 to every participant, so the white-bread condition is completely confounded with a first-period effect: any systematic difference between the first and the later sessions—familiarization with the procedure, anticipatory stress, or day-to-day variation in habitual intake and activity—is statistically indistinguishable from a true effect of white bread. This affects every contrast against the reference, including the primary total iAUC contrast, and it cannot be corrected analytically; it is the main reason why the present results should be regarded as provisional. Further limitations are the small sample size, nonrandom sequence assignment, snowball recruitment, the narrow observed age range of 21–25 years, and a BMI range extending into the overweight category (19.7–29.5 kg/m^2^), which may increase intra-individual variability in postprandial responses [27]. White bread was always administered first; thus, its effect cannot be separated from a first-period effect. Its session also had the lowest mean fasting glucose, which may have influenced baseline-referenced contrasts despite the nonsignificant overall baseline test. Comparisons among the four test breads were not subject to this specific first-period confounding, and the purple–yellow-durum difference had the same direction in both sequences. Nevertheless, incomplete counterbalancing makes residual period and sequence effects possible. The study was also not prospectively registered in a public trial registry. Eligibility relied on self-reported health, and the preceding evening meal, adherence variables, and menstrual phase were not controlled or formally recorded. Smoking status and habitual alcohol consumption were not recorded, thereby limiting the assessment of their potential influence on postprandial glycemic responses. Individual biological and measurement variability further limits precision in a small acute study [9,10,27,28].

The home setting and absence of participant blinding limited experimental control. Using a single glucose reading per time point and omitting the 15 and 45 min samples can obscure peak magnitude and timing [29]. The fresh yeast-to-flour ratio, nominal resting, final proofing, and baking conditions were standardized across formulations. However, internal bread temperature was not measured, and flour-specific differences in water absorption and dough properties may have influenced fermentation and final bread structure. Therefore, the common preparation conditions do not establish equivalent starch gelatinization or bread structure across formulations, thereby limiting the attribution of the observed glycemic differences to flour composition alone. Available carbohydrate doses were estimated from manufacturer declarations rather than analytically confirmed in the served breads. Thus, the exact carbohydrate equivalence across conditions, including the white-bread reference, remains uncertain. The undeclared dietary fiber content of the white reference flour also hindered complete comparison of fiber doses.

Future studies should use randomized, fully counterbalanced crossover designs, repeated reference administrations, and served portions based on analytically confirmed available carbohydrate. Larger samples and more closely standardized preparation and measurement conditions would help confirm the observed formulation differences and evaluate their generalizability.

## 5. Conclusions

In this small, exploratory, nonrandomized within-participant study, the tested purple-flour formulation was associated with lower early postprandial glycemic exposure than white bread and each of the other three test breads; this early-phase difference was the most consistent finding and was supported by both the parametric and the rank-based analyses. Its primary difference in total iAUC compared with white bread was statistically significant only in the parametric analysis and should therefore be regarded as provisional. The lower total iAUC relative to yellow durum bread emerged from exploratory comparisons and should not be afforded the same confidence as the prespecified reference comparison. For Karakılçık bread, no statistically significant lower acute response was detected under the tested conditions; given the small sample and the wide confidence intervals, this should not be interpreted as evidence of no effect. Notably, the two wholemeal flours with the highest declared fiber content did not produce the lowest responses, so a higher declared fiber content was not consistently associated with lower acute glycemic responses in the products tested. These formulation-specific findings apply only to the particular commercial products tested and should not be generalized to purple wheat, einkorn, or Karakılçık as grain categories. Confirmation requires randomized, fully counterbalanced studies with analytically verified composition and repeated reference administrations.

## Figures and Tables

**Table 1:** Product information for the five commercial flours, as declared by the manufacturer.

Attribute	White Bread (Reference)	Purple Flour	Einkorn	Yellow Durum	Karakılçık
Manufacturer	Nora Un, Ankara, Türkiye	Nora Un, Ankara, Türkiye	Nora Un, Ankara, Türkiye	Nora Un, Ankara, Türkiye	Nora Un, Ankara, Türkiye
Label designation	Wheat flour	Special purpose wheat flour	Wholemeal einkorn flour	Special purpose wheat flour (durum)	Wholemeal Karakılçık flour
Batch/lot number	[lot no.]	[lot no.]	[lot no.]	[lot no.]	[lot no.]
Wheat species	*T. aestivum* L.	*T. aestivum* L.	*T. monococcum* L.	*T. turgidum* subsp. *durum*	*T. turgidum* subsp. *durum*
Cultivar or landrace	ND	ND	ND	ND	Karakılçık landrace
Milling status	Refined	Refined	Wholemeal	Refined	Wholemeal
Extraction (ash) rate	ND	ND	ND	ND	ND
Declared ingredients	Wheat flour	Wheat flour; ascorbic acid (E-300); extracts of blackberry, sour purple mulberry, blueberry, pomegranate, sour cherry, eggplant, and purple onion skin	Wholemeal einkorn flour; ascorbic acid (E-300)	Durum wheat flour; ascorbic acid (E-300)	Wholemeal Karakılçık flour; ascorbic acid (E-300)
Ascorbic acid (E-300)	Not declared	Declared; amount ND	Declared; amount ND	Declared; amount ND	Declared; amount ND
Added plant extracts	None declared	7 extracts; amounts ND	None declared	None declared	None declared
Other declared additives	None	None	None	None	None

ND, not disclosed by the manufacturer, meaning that the item was neither printed on the label nor supplied by the manufacturer on request. All information was taken from the product labels and from the manufacturer’s product information available at the time of the study; declared nutrient values for the same products are given in Table 2. No compositional analysis of the flours or of the baked breads was performed by the research team.

**Table 2:** Recipe inputs and pre-baking dough-portion calculations.

Bread	Flour (g)	Water (mL)	Water-to-Flour Ratio	Fresh Yeast (g; % Flour)	Salt (g; % Flour)	Pre-Baking Recipe Mass (g)	Calculated Pre-Baking Dough Portion (g)	Label-Derived avCHO (g)
White bread (reference)	600	350.0	58.3	25.2 (4.2)	6 (1.0)	981.20	115.98	50.0
Purple flour	600	350.0	58.3	25.2 (4.2)	6 (1.0)	981.20	116.81	50.0
Einkorn	500	291.7	58.3	21.0 (4.2)	6 (1.2)	818.70	116.96	50.0
Yellow durum	575	335.4	58.3	24.2 (4.2)	6 (1.0)	940.55	116.84	50.0
Karakılçık	500	291.7	58.3	21.0 (4.2)	6 (1.2)	818.70	116.96	50.0

avCHO, available carbohydrate. Recipe mass was calculated assuming a 1 g/mL water density.

**Table 3:** Manufacturer-declared flour composition and calculated flour-derived nutrients per portion. All values are manufacturer label declarations and were not verified by laboratory analysis.

Bread	Milling	kcal/100 g	CHO/100 g	Fiber/100 g	Prot./100 g	Fat/100 g	avCHO/port.	Fiber/port.	Prot./port.	kcal/port.
White bread (reference)	Refined	347	70.5	NR	10.0	1.7	50.0	NR	7.1	246
Purple flour	Refined	358	70.0	2.03	12.0	0.8	50.0	1.5	8.6	256
Einkorn	Whole	327	70.0	13.0	12.0	1.2	50.0	9.3	8.6	234
Yellow durum	Refined	350	70.0	2.03	13.0	2.0	50.0	1.5	9.3	250
Karakılçık	Whole	350	70.0	13.0	12.0	1.0	50.0	9.3	8.6	250

Columns 3–7 are per 100 g flour as declared by the manufacturer; columns 8–11 are calculated from the flour contained in one study portion. CHO, carbohydrate; avCHO, available carbohydrate; Prot., protein; NR, not reported. Carbohydrate declarations excluded dietary fiber. None of the values in this table were measured analytically, either in the flours or in the baked breads.

**Table 4:** Participant characteristics (*n* = 12).

Characteristic	Mean ± SD
Age (years)	22.7 ± 1.2
Height (cm)	173.5 ± 6.8
Body weight (kg)	68.9 ± 13.9
BMI (kg/m^2^)	22.7 ± 2.8
Waist circumference (cm)	82.8 ± 12.2
Hip circumference (cm)	101.2 ± 6.1
Waist-to-hip ratio	0.81 ± 0.07
Fasting capillary glucose (mg/dL) ^1^	95.1 ± 5.6

SD, standard deviation; BMI, body mass index. Height ranged from 165 to 186 cm and BMI from 19.7 to 29.5 kg/m^2^. ^1^ Mean of the five session-specific fasting values.

**Table 5:** Glycemic response outcomes by bread type (*n* = 12).

Bread	Total iAUC 0–120 min (mg·min/dL)	Early iAUC 0–60 min (mg·min/dL)	*C*_max_ (mg/dL)	*T*_max_ (min), Median [IQR]	Fasting Glucose (mg/dL)
White bread (reference)	3023.2 ± 985.8	1826.2 ± 416.1	46.3 ± 8.5	30 [30–30]	93.5 ± 6.8
Purple flour	2051.0 ± 998.0	1238.8 ± 492.0	35.1 ± 11.2	30 [30–37.5]	96.1 ± 6.1
Einkorn	2500.0 ± 1065.6	1558.8 ± 477.3	41.1 ± 11.8	30 [30–37.5]	94.8 ± 7.3
Yellow durum	3370.8 ± 511.5	2052.5 ± 392.6	51.0 ± 9.6	30 [30–30]	95.2 ± 5.3
Karakılçık	3069.3 ± 930.3	1787.5 ± 344.5	46.1 ± 12.2	30 [30–30]	96.2 ± 7.7

Values are expressed as mean ± SD unless otherwise stated. IQR, interquartile range; iAUC, incremental area under the curve. Total iAUC, early iAUC, *C*_max_, and fasting glucose were analyzed via one-way repeated-measures ANOVA with Greenhouse–Geisser correction and *T*_max_ via the Friedman test.

**Table 6:** Primary (total iAUC) and secondary (early iAUC and *C*_max_) contrasts between each test bread and the white-bread reference (*n* = 12).

Outcome	Contrast	Mean Difference [95% CI]	Holm *p* (*t*-Test)	Holm *p* (Wilcoxon)	Cohen’s *d*_z_
Total iAUC (0–120 min)	Purple flour—White	−972.2 [−1645.0, −299.4]	**0.035**	0.064	−0.92
	Einkorn—White	−523.2 [−1481.9, 435.6]	0.765	0.798	−0.35
	Yellow durum—White	347.6 [−360.6, 1055.8]	0.765	0.798	0.31
	Karakılçık—White	46.1 [−659.4, 751.6]	0.888	0.970	0.04
Early iAUC (0–60 min)	Purple flour—White	−587.5 [−1005.4, −169.6]	**0.041**	**0.049**	−0.89
	Einkorn—White	−267.5 [−760.7, 225.7]	0.773	0.798	−0.34
	Yellow durum—White	226.2 [−229.8, 682.3]	0.773	0.798	0.32
	Karakılçık—White	−38.8 [−370.8, 293.3]	0.802	0.805	−0.07
*C*_max_	Purple flour—White	−11.2 [−20.4, −2.1]	0.082	0.109	−0.78
	Einkorn—White	−5.2 [−16.6, 6.1]	0.985	0.472	−0.29
	Yellow durum—White	4.7 [−5.4, 14.7]	0.985	0.842	0.30
	Karakılçık—White	−0.2 [−8.5, 8.0]	0.985	0.895	−0.02

CI, confidence interval; iAUC, incremental area under the curve. Four two-sided paired-sample *t*-tests were conducted per outcome and adjusted using Holm’s method within that outcome. Wilcoxon signed-rank tests were sensitivity analyses with the same adjustment. iAUC is expressed in mg·min/dL and *C*_max_ in mg/dL. Values in bold are statistically significant following Holm adjustment (*p* < 0.05). The total iAUC contrasts constitute the primary analysis and the early iAUC and *C*_max_ contrasts the secondary analyses. Confidence intervals are nominal and were not adjusted for multiplicity; they may therefore exclude zero when the Holm-adjusted *p*-value is ≥0.05, and the Holm-adjusted *p*-value takes precedence for inference.

**Table 7:** Exploratory pairwise comparisons among the four test breads (*n* = 12).

Outcome	Contrast	Mean Difference [95% CI]	Holm *p* (*t*-Test)	Holm *p* (Wilcoxon)	Cohen’s *d*_z_
Total iAUC	Purple—Einkorn	−449.0 [−1030.0, 131.9]	0.351	0.330	−0.49
Total iAUC	Purple—Yellow durum	−1319.8 [−2050.3, −589.4]	**0.013**	**0.032**	−1.15
Total iAUC	Purple—Karakılçık	−1018.3 [−1784.6, −252.0]	0.066	0.107	−0.84
Total iAUC	Einkorn—Yellow durum	−870.8 [−1520.2, −221.4]	0.066	0.063	−0.85
Total iAUC	Einkorn—Karakılçık	−569.3 [−1350.5, 211.9]	0.351	0.330	−0.46
Total iAUC	Yellow durum—Karakılçık	301.5 [−306.2, 909.2]	0.351	0.424	0.32
Early iAUC	Purple—Einkorn	−320.0 [−515.1, −124.9]	**0.016**	**0.027**	−1.04
Early iAUC	Purple—Yellow durum	−813.8 [−1126.5, −501.0]	**<0.001**	**0.006**	−1.65
Early iAUC	Purple—Karakılçık	−548.8 [−890.7, −206.8]	**0.016**	**0.027**	−1.02
Early iAUC	Einkorn—Yellow durum	−493.8 [−698.5, −289.0]	**0.001**	**0.010**	−1.53
Early iAUC	Einkorn—Karakılçık	−228.8 [−555.9, 98.4]	0.158	0.135	−0.44
Early iAUC	Yellow durum—Karakılçık	265.0 [−36.3, 566.3]	0.158	0.135	0.56
*C*_max_	Purple—Einkorn	−6.0 [−12.6, 0.6]	0.209	0.265	−0.58
*C*_max_	Purple—Yellow durum	−15.9 [−24.2, −7.6]	**0.008**	**0.023**	−1.22
*C*_max_	Purple—Karakılçık	−11.0 [−19.8, −2.2]	0.074	0.105	−0.80
*C*_max_	Einkorn—Yellow durum	−9.9 [−15.8, −4.1]	**0.017**	**0.034**	−1.08
*C*_max_	Einkorn—Karakılçık	−5.0 [−15.4, 5.4]	0.472	0.365	−0.31
*C*_max_	Yellow durum—Karakılçık	4.9 [−3.7, 13.6]	0.472	0.365	0.36

CI, confidence interval; iAUC, incremental area under the curve. Six paired-sample *t*-tests were adjusted using Holm’s method for each outcome; Wilcoxon signed-rank tests were subjected to the same adjustment. iAUC is expressed in mg·min/dL and *C*_max_ in mg/dL. Values in bold are statistically significant following Holm adjustment (*p* < 0.05). All comparisons in this table are exploratory and hypothesis-generating; none was prespecified as a confirmatory analysis, and they should not be given the same evidentiary weight as the primary and secondary contrasts in Table 6. Confidence intervals are nominal and unadjusted for multiplicity.

## Data Availability

The participant-level data supporting the findings of this study are available from the corresponding author upon reasonable request. The data are not publicly available because they were collected under a consent procedure that did not include a public deposition.

## Abbreviations

The following abbreviations are used in this manuscript:

ANOVA	Analysis of variance
avCHO	Available carbohydrate
BMI	Body mass index
CHO	Carbohydrate
CI	Confidence interval
C_max_	Peak incremental glucose concentration
GI	Glycemic index
iAUC	Incremental area under the curve
IQR	Interquartile range
ISO	International Organization for Standardization
NR	Not reported
SD	Standard deviation
T_max_	Time to peak incremental glucose concentration
η_p_^2^	Partial eta squared
